# Inhibition of dipeptidyl peptidase 8/9 impairs preadipocyte differentiation

**DOI:** 10.1038/srep12348

**Published:** 2015-08-05

**Authors:** Ruijun Han, Xinying Wang, William Bachovchin, Zofia Zukowska, John W. Osborn

**Affiliations:** 1 Department of Integrative Biology and Physiology, University of Minnesota, Minneapolis, MN, USA; 2 Sackler School of Biomedical Sciences, Tufts University School of Medicine, Boston, MA, USA

## Abstract

Adipocytes are the primary cells in adipose tissue, and adipocyte dysfunction causes lipodystrophy, obesity and diabetes. The dipeptidyl peptidase (DPP) 4 family includes four enzymes, DPP4, DPP8, DPP9 and fibroblast activation protein (FAP). DPP4 family inhibitors have been used for the treatment of type 2 diabetes patients, but their role in adipocyte formation are poorly understood. Here we demonstrate that the DPP8/9 selective inhibitor 1G244 blocks adipogenesis in preadipocyte 3T3-L1 and 3T3-F422A, while DPP4 and FAP inhibitors have no effect. In addition, knockdown of DPP8 or DPP9 significantly impairs adipocyte differentiation in preadipocytes. We further uncovered that blocking the expression or activities of DPP8 and DPP9 attenuates PPARγ2 induction during preadipocyte differentiation. Addition of PPARγ agonist thiazolidinediones (TZDs), or ectopic expression of PPARγ2, is able to rescue the adipogenic defect caused by DPP8/9 inhibition in preadipocytes. These results indicate the importance of DPP8 and DPP9 on adipogenesis.

Over the last several decades, increase in the prevalence of maladies such as metabolic disease, cardiovascular diseases, and even some cancers is often attributed to the paralleled rise in obesity rate worldwide^1^,^2^,^3^. The obesity phenotype is often characterized by the increase in fat mass, or adipose tissue accumulation. This adipose tissue is primarily comprised of adipocytes, which store excess energy as triglycerides and secrete various endocrine signals, which in turn and contributes to the regulation of total body energy homeostasis^4^,^5^,^6^,^7^. While the role of adipose tissue in obesity has been thoroughly examined, the development and proliferation of adipocytes remain an important question in obesity research. To this end, further examination of adipocyte development is important for the treatment and prevention of obesity and associated diseases.

Much of our knowledge on adipogenesis comes from *in vitro* studies of fibroblasts or pre-adipocytes, such as the mouse cell lines 3T3-L1, 3T3-F442A^4^. Adipocyte differentiation is coordinated by the nuclear receptor peroxisome proliferator-activated receptor γ (PPARγ) and members of the CCAAT enhancer binding proteins (CEBPs) family^8^. Many of the genes involved in adipogenesis are targets of PPARγ and/or CEBPα^9^,^10^,^11^. Thiazolidinediones (TZDs), which are known to have potent adipogenic and antidiabetic effects, are agonists for PPARγ. TZDs promote adipogenesis and adipokine production in adipose tissue^8^,^12^.

Proteases of the S9B/dipeptidyl peptidase (DPP) 4 family are serine amino peptidases. They comprise of four enzymes: DPP4, fibroblast activation protein (FAP), DPP8 and DPP9. Active members of the DPP4 family preferentially cleave Xaa-Pro- and Xaa-Ala- dipeptides (where Xaa is any amino acid except proline) from the N-terminus of proteins^13^. Further, DPP4 and FAP are cell surface peptidases, and substrates of DPP4 include numerous neuropeptides, hormones and chemokines, such as: fibronectin, substance P, neuropeptide Y, peptide YY , glucagon-like-peptides 1 and 2 (GLP-1, GLP-2) and glucose-dependent insulinotropic peptide (GIP)^13^. Particularly, two substrates of DPP4, GLP-1 and GIP, are released from the intestinal mucosa and account for ∼60% of postprandial insulin secretion^14^,^15^. Inhibition of DPP4 prolongs the activity of GLP and GIP, playing an important role in insulin secretion and blood glucose regulation^16^,^17^. Therefore, DPP4 inhibitors have drawn increased attention, and are in clinical use as antidiabetic drugs^13^,^17^.

Mice lacking DPP4 or mice treated with DPP4 inhibitors are resistant to high fat diet (HFD) induced obesity and insulin resistance^18^,^19^. These results were attributed to the reduced food intake and increased energy expenditure in the HFD-treated mice^18^,^19^. Human diabetic subjects treated with DPP4 inhibitors show a significant decrease in HbA1c, postprandial glucose, and circulating triglyceride levels compared to placebo treatment group^20^. Interestingly, despite their wide use in treating type 2 diabetes, the importance of DPP4 family inhibitors on adipogenesis is yet to be identified.

The two cytosolic members of the DPP4 family are DPP8 and DPP9 that share approximately 60% homology^21^,^22^. *In vitro,* DPP8 and DPP9 are similar in their biochemical properties, including enzyme kinetics and substrate specificity^23^,^24^. However, the importance of DPP8 and DPP9 in obesity and diabetes remain undetermined.

In the present study, we examined the effect of three DPP specific inhibitors on adipocyte differentiation in preadipocytes: DPP4-selective inhibitor MK-0431, DPP8/9-selective inhibitor 1G244, and the FAP-selective inhibitor 3099. We found that only the DPP8/9 inhibitor 1G244 blocked adipogenesis in preadipocytes. We further discovered that inhibition of DPP8 and DPP9 attenuated the expression of PPARγ2 in basal level and during preadipocyte differentiation.

## Results

### DPP4 family inhibitor P32/98 impairs adipocyte differentiation

**Figure 1:**
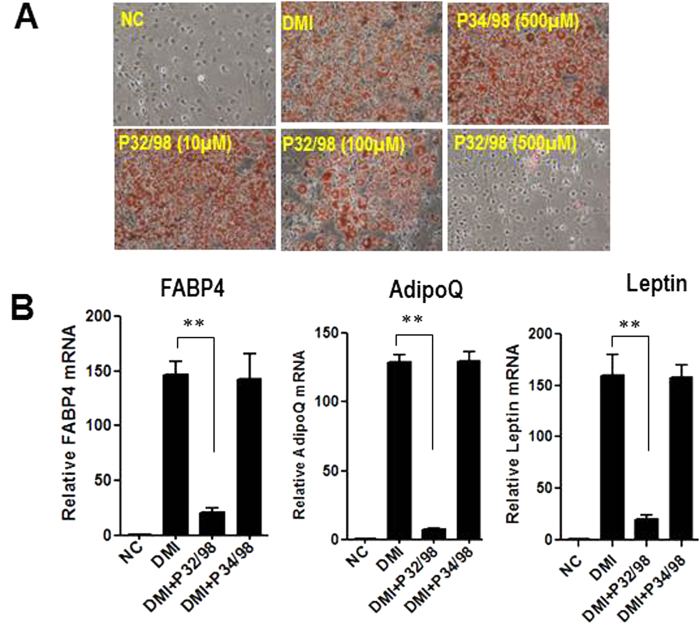
The non-specific DPP4 family inhibitor P32/98 impairs adipocyte differentiation. (**A**) Oil Red O staining of control 3T3-L1 cells (DMI) and 3T3-L1 cells treated with DPP4 family inhibitor P32/98 (P32/98) or inactive inhibitor (P34/98) at day 8 of differentiation. (**B**) Adipocyte markers, FABP4, adiponectin (AdipoQ) and leptin, were measured by real time PCR at day 8 of differentiation. β-actin expression was used as an internal control.

To address whether the DPP4 family (DPP4, DPP8, DPP9 and FAP) play a role in preadipocyte differentiation, we added the non-selective competitive DPP4 family inhibitor isoleucine-thiazolidide (P32/98) to the classic 3T3-L1 differentiation cocktail: dexamethasone, methylxanthine and insulin (DMI). We found that P32/98 blocked adipogenesis in a dose dependent manner, starting at the concentration of 100 μM, as assessed by oil red O staining (Fig. 1A). At 500 μM concentration, P32/98 completely blocked adipogenesis in 3T3-L1 cell line. However, 500 μM concentration of a chemically similar but non-inhibitory compound P34/98 had no effect (Fig. 1A). We further confirmed the inhibitory effects of P32/98 by detecting the expression of adipocyte markers at the end of differentiation. The expression of FABP4, adiponectin and leptin was markedly decreased in 3T3-L1 adipocytes when treated with P32/98 during adipogenesis (Fig. 1B). A similar inhibitory effect of P32/98 on adipocyte differentiation was observed in 3T3-F442A preadipocytes (supplementary Fig. 1). Notably, only the high dose (over 100 μM) of P32/98 blocked adipogenesis in preadipocytes. Since P32/98 has IC50 values of 460 nM for DPP4, 2180 nM for DPP8 and 1600 nM for DPP9^25^, we suggest that the activities of DPP4, DPP8 and DPP9 were all inhibited in preadipocytes.

### DPP8/9 inhibition blocks adipocyte differentiation

**Figure 2:**
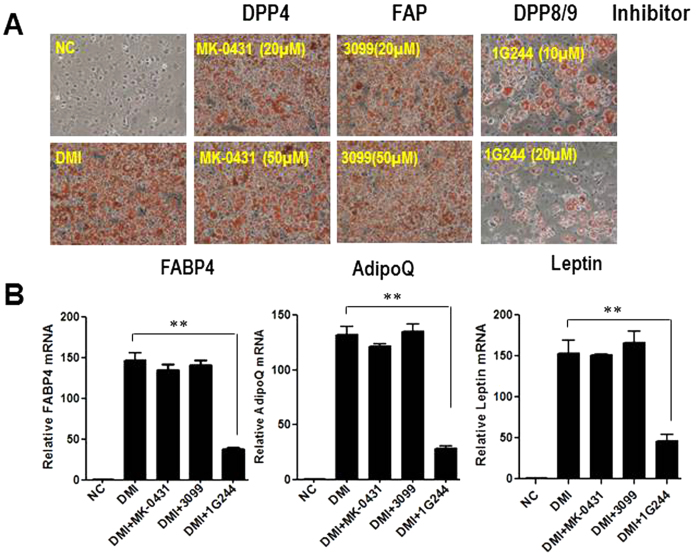
DPP8/9 inhibitor impairs adipocyte differentiation. (**A**) Oil Red O staining of 3T3-L1 control cells (DMI) and cells treated with specific DPP inhibitors like DPP4 inhibitor MK-0431(DMI+ MK-0431), the DPP8/9 inhibitor 1G244 (DMI+1G244) and FAP inhibitor 3099 (DMI+3099) at day 8 of differentiation. (**B**) Adipocyte markers, FABP4, adiponectin (AdipoQ) and leptin were measured by real time PCR at day 8 of differentiation. β-actin expression was used as an internal control.

To further investigate which DPP contributes to the adipogenic defect as described above, specific DPP inhibitors such as a DPP4-selective inhibitor MK-0431/Sitagliptin, DPP8/9-selective inhibitor 1G244, and FAP-selective inhibitor 3099, were added during 3T3-L1 differentiation. We found that the DPP4 inhibitor MK-0431 and the FAP inhibitor 3099 had no effect on adipocyte differentiation, while the DPP8/9 inhibitor 1G244 blocked adipogenesis in 3T3-L1 cells as measured by Oil red O staining (Fig. 2A). At the end of differentiation, the expression of the adipocyte markers, FABP4, adiponectin and leptin, were markedly decreased (Fig. 2B) after treatment with the DPP8/9 inhibitor 1G244. No difference in the expression of these markers was found with either DPP4 inhibitor MK-0431 or FAP inhibitor 3099 (Fig. 2B). A similar inhibitory effect of the DPP8/9 inhibitor 1G244 on adipogenesis was observed in 3T3-F442A preadipocytes (supplementary Fig. 1). Taken together, these data indicate that DPP8/9, but not DPP4 or FAP, is essential for adipogenesis.

### Time dependent expression of DDP4 family during 3T3-L1 differentiation

**Figure 3:**
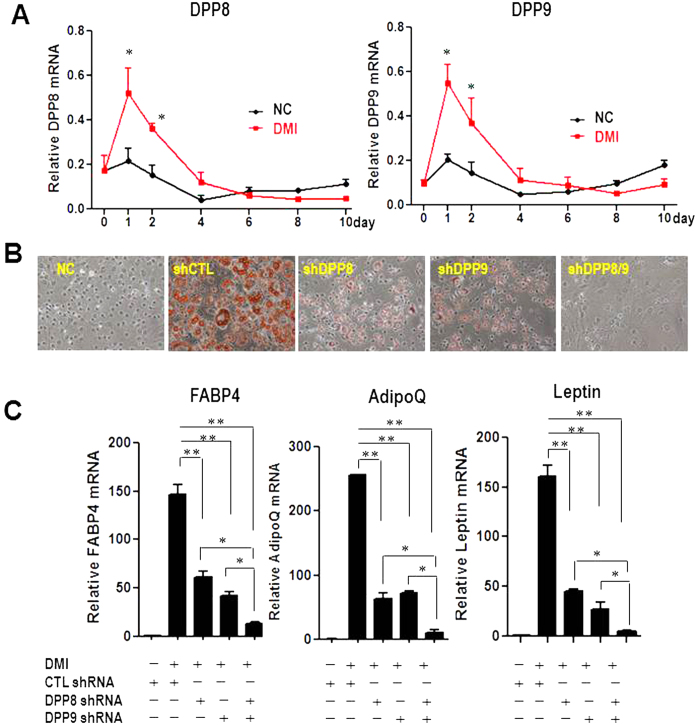
Knockdown of DPP8 and DPP9 blocked adipocyte differentiation. (**A**) Gene expression analysis of DPP8 and DPP9 during 3T3L1 differentiation. 3T3L1 cells were cultured in 10% FBS (NC) or treated with dexamethasone, isobutylmethylxanthine and insulin (DMI) from day 0 to day 2 of differentiation, mRNA levels of DPP8 and DPP9 were measured by real time PCR. β-actin expression was used as an internal control. (**B**) Oil Red O staining of control-shRNA-transduced cells (shCTL) and cells with DPP8/9 shRNAs (shDPP8, shDPP9) at day 8 of differentiation. (**C**) Adipocyte markers, FABP4, adiponectin (AdipoQ) and leptin, were measured by real time PCR at day 8 of differentiation. β-actin expression was used as an internal control.

We next assessed the gene expression and enzyme activity of DPPs during adipocyte differentiation. The mRNA level of DPP4 and FAP were low after 48 hours of DMI treatment, whereas the DPP8 and DPP9 mRNA levels were much higher (supplementary Fig. 2A). Evidence suggest that majority of the DPPs activity during differentiation is through DPP8/9 as the DPP8/9 inhibitor 1G244 blocked 82% of total DPPs activity in 3T3-L1 cells; while MK-0431 blocked only 15% and 3099 blocked 20%, respectively (supplementary Fig. 2B). We tested whether DPP8/9 was a regulated component of the differentiation program and if its expression changed over the course of differentiation. Indeed, analysis of a time course of 3T3-L1 cell differentiation revealed that DPP8/9 mRNA expression was significantly increased during DMI treatment (Fig. 3A). Conversely, DPP4 and FAP mRNA expression were decreased (supplementary Fig. 3).

### DPP8 and DPP9 are required for 3T3-L1 differentiation

To further validate the adipogenic action of DPP8 and DPP9, we generated stable cell lines with knockdown of DPP8, DPP9 or both using shRNAs. The efficiency and specificity of DPP8 or DPP9 knockdown was confirmed by real time PCR and western blot (Supplementary Fig. 4). Stable 3T3-L1 cell lines expressing shRNA sequences targeting DPP8 or DPP9 exhibited reduced differentiation capacity, compared to those expressing a control shRNA, as assessed by oil red O staining (Fig. 3B). Knockdown of both DPP8 and DPP9 completely blocked adipocyte differentiation (Fig. 3B). In agreement with the morphological differentiation, the expression of the adipocyte markers FABP4, adiponectin and leptin were also impaired in DPP8, DPP9 and DPP8/9 knockdown cells (Fig. 3C).

**Figure 4:**
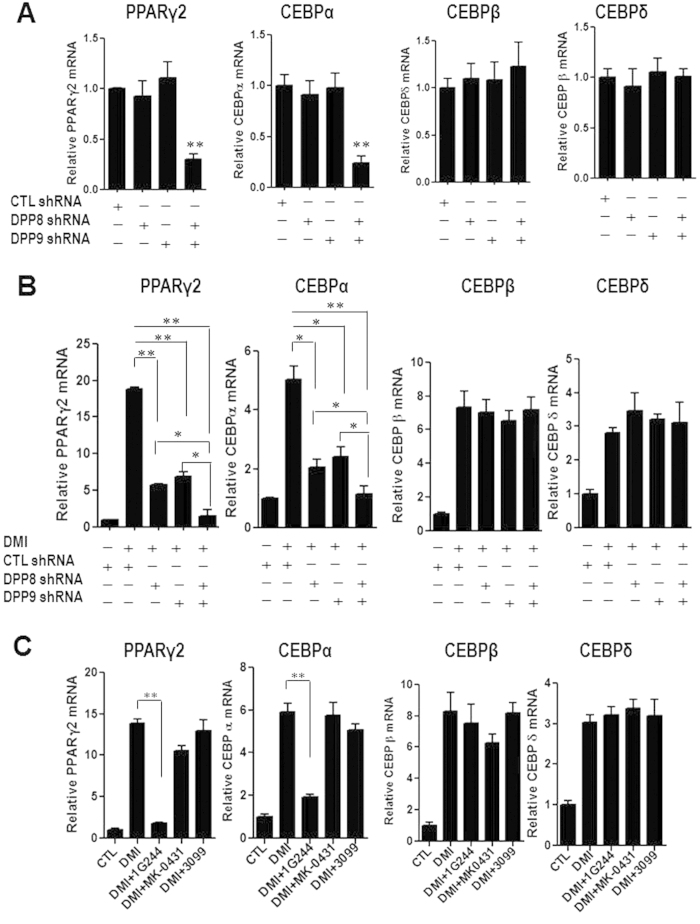
Inhibition of DPP8 and DPP9 prevents PPARγ2 induction during 3T3-L1 differentiation. (**A**) PPARγ2, CEBPα, CEBPβ and CEBPδ mRNA levels were measured in control-shRNA-transduced 3T3-L1 cells (shCTL) and cells with DPP8/9 shRNAs (shDPP8, shDPP9) at basal level. (**B**) PPARγ2 and CEBPα mRNA levels were measured in control-shRNA-transduced cells (shCTL.) and cells with DPP8/9 shRNAs (shDPP8, shDPP9) after 48 hour treatment of DMI. CEBPβ and CEBPδ mRNA levels were measured after 6 hour treatment. (**C**) PPARγ2 and CEBPα mRNA levels were measured after 48 hour treatment of dexamethasone, isobutylmethylxanthine and insulin (DMI) with or without the DPP4 inhibitor MK-0431(DMI+MK-0431), the DPP8/9 inhibitor 1G244 (DMI+1G244), the FAP inhibitor 3099 (DMI+3099). CEBPβ and CEBPδ mRNA levels were measured in these cells after 6 hour treatment.

Next we sought to elucidate the mechanism of action of DPP8/9 in preadipocyte differentiation. PPARγ is the master regulator of adipocyte differentiation. Activation of PPARγ at an early stage is necessary and sufficient for adipogenesis^4^. We hypothesized that the effect of DPP8/9 inhibitor on adipogenesis was mediated by PPARγ. The expression PPARγ2 was significantly decreased in stable 3T3-L1 cell expressing shRNA sequences targeting both DPP8 and DPP9, compared to those expressing a control shRNA at basal level with no differentiation cocktail (DMI) (Fig. 4A). When treated with a differentiation cocktail DMI, PPARγ2 was markedly increased after 48 hours in 3T3-L1 cells (Fig. 4B). DMI induced expression of PPARγ2 was partly blocked with depletion of either DPP8 or DPP9. Since PPARγ gene is regulated by CEBPs^4^, we determined the effect of DPP8 or DPP9 depletion on the expression of CEBPα, CEBPβ and CEBPδ. We found that depletion of DPP8 or DPP9 also attenuated basal and DMI-induced CEBPα mRNA levels (Fig. 4A,B) but had no effect on expression of CEBPβ and CEBPδ (Fig. 4A,B). Knockdown of both DPP8 and DPP9 completely blocked the DMI induced expression of PPARγ2 and CEBPα (Fig. 4B) in 3T3-L1. In addition, the DPP8/9 inhibitor 1G244 attenuated DMI-induced PPARγ2 and CEBPα mRNA (Fig. 4C), but had no effect on the expression of CEBPβ and CEBPδ (Fig. 4C). On the other hand, DPP4 inhibitor MK-0431 and FAP inhibitor 3099 had no effect on DMI induced expression of CEBPα, CEBPβ, CEBPδ and PPARγ2 during 3T3-L1 differentiation (Fig. 4C).

### TZDs or ectopic PPARγ2 rescues inhibition of DPP8/9 induced adipogenic defects

**Figure 5:**
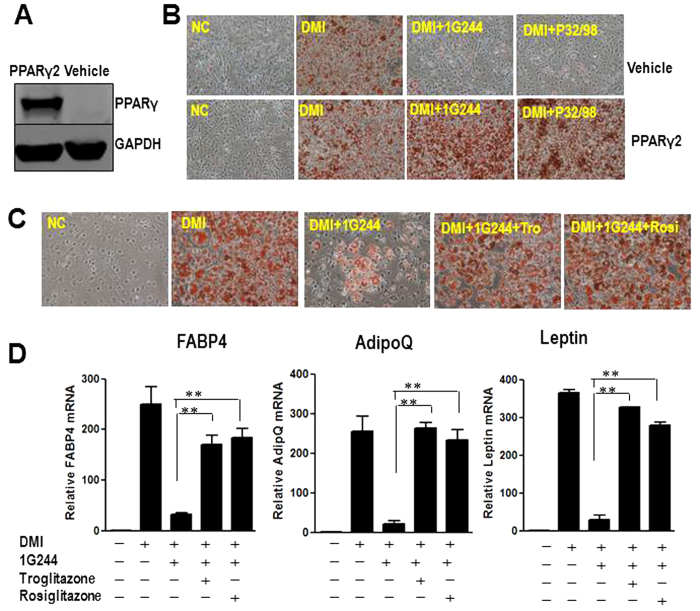
TZDs or ectopic PPARγ2 rescues inhibition of DPP8/9 induced adipogenic defects in 3T3-L1 cells. (**A**) Representative western blot for the expression of PPARγ in stable 3T3-L1 cells transduced with control plasmid (vehicle) or PPARγ2 plasmid (PPARγ2). The blots were cropped, and the full-length blots are presented in the supplementary information. (**B**) Oil Red O staining of control cells or PPARγ2 overexpressed 3T3-L1 cells, treated vehicle (NC), DMI (DMI), 500 μM non-selective DPP4 family inhibitor P32/98 (DMI+P32/98) or 20 μM DPP8/9 inhibitor 1G244 (DMI+1G244) at day 8 of differentiation. (**C**) Oil Red O staining of 3T3-L1 cells treated with 20μM DPP8/9 inhibitor 1G244 (DMI+1G244) or 1G244 plus 1 μM rosiglitazone(DMI+1G244+Rosi) or 5 μM troglitazone (DMI+1G244+Tro). (**D**) Adipocyte markers, FABP4, adiponectin (AdipoQ) and leptin, were measured in these cells by real time PCR at day 8 of differentiation. β-actin expression was used as an internal control.

To determine whether PPARγ2 can rescue the adipogenic defect caused by DPP8/9 inhibition, we generated stable PPARγ2 transduced 3T3-L1 cells (Fig. 5A). Ectopic expression of PPARγ2 was able to rescue P32/98 or 1G244 caused adipogenic defect in preadipocytes (Fig. 5B). The TZDs, rosiglitazone or troglitazone, are known ligands of PPARγ. To investigate whether rosiglitazone or troglitazone were able to rescue the adipogenic defect in DPP8/9 inactivated preadipocytes, we added the DPP8/9 inhibitor 1G244 with either rosiglitazone or troglitazone during 3T3-L1 differentiation. In the absence of rosiglitazone or troglitazone, 1G244 markedly inhibited the adipognesis in 3T3-L1 cells. However, administration of the rosiglitazone or troglitazone completely rescued the adipognesis defect caused by 1G244, as assessed by oil red O staining (Fig. 5C) and confirmed by the expression of adipocyte markers FABP4, adiponectin and leptin (Fig. 5D).

## Discussion

Compared to the well-studied DPP4, the functions of DPP8 and DPP9 are mostly unclear. To date, there are several studies indicating that DPP8 and DPP9 are involved in immune responses and cancer biology^26^,^27^,^28^. Here, for the first time, we report that DPP8 and DPP9 are indispensable for adipocyte differentiation. We show that DPP8 and DPP9 mRNA are significantly increased during adipocyte differentiation and DPP8/DPP9 knockdown impairs preadipocyte differentiation. We have evaluated the expression of both DPP8 and DPP9 by immunoblotting using commercially available antibodies. We succeeded to detect only the endogenous DPP8 protein in preadipocytes (Supplementary Fig. 4). However, after trying three DPP9 antibodies from different venders, we have not found a suitable antibody to detect the endogenous DPP9 protein in 3T3-L1 cells. Although it is difficult to determine the natural endogenous substrate of DPP8 and DPP9 that mediate adipogenesis, there is evidence in the literature to support a potential biological role of DPP8/DPP9 in metabolism^29^. DPP8 and DPP9 are homologous, cytoplasmic N-terminal post-proline-cleaving enzymes. To this day, more than 29 *in vivo* DPP8 and DPP9 substrate candidates have been discovered using terminal amine isotopic labeling of substrates technique^29^. Most of these substrates are involved in regulating cellular metabolism and energy homeostasis, suggesting potential roles for DPP8 and DPP9 in cellular metabolic pathways, including glycolysis, gluconeogenesis, fatty acid metabolism, and nucleotide metabolism/biosynthesis^29^. The future goal will be to identify the substrates of DPP8/9 mediating preadipocyte differentiation.

PPARγ is a member of the nuclear-receptor superfamily and is both necessary as well as sufficient for adipogenesis^4^. The relative roles of PPARγ1 and PPARγ2 in adipogenesis remain an open question. Both PPARγ1 and PPARγ2 are induced during adipogenesis, but PPARγ1 is also expressed in macrophages, liver and skeletal muscle and colonic epithelium^4^. In addition, PPARγ2 is highly induced during differentiation and is critical in promoting adipogenesis in preadipocytes^30^,^31^. In our study, DPP8/9 inhibitor reduced both PPARγ1 and PPARγ2 induction during adipocyte differentiation (Supplementary Figure 5). Consistent with previous reports, we have found that PPARγ2 is highly upregulated during 3T3-L1 differentiation. Therefore, we and others^32^ have selected PPARγ2 as a marker for preadipocyte differentiation.

PPARγ regulates adipogenesis together with members of the CEBP family. CEBPβ and CEBPδ are expressed early during preadipocyte differentiation^33^,^34^, and are involved in the induction of PPARγ and CEBPα. CEBPα and PPARγ are induced at later stages and are also active in mature adipocytes^4^. Despite the importance of CEBPs, CEBPs are not sufficient to induce adipogenesis in the absence of PPARγ. Some transcription factors, like KLF15, KLF5, GATA2 and GATA 3 directly target the expression of PPARγ and CEBPα, with no effects on the expression of CEBPβ and CEBPδ during the adipogenesis^4^. Similarly, here we also find that inhibition of DPP8 and DPP9 successfully attenuated the induction of PPARγ and CEBPα, with no effects on CEBPβ and CEBPδ. The activation of CEBPα and PPARγ are partly dependent on the expression of CEBPβ and CEBPδ. For example, transcription factor KLF15 induces the expression PPARγ during adipocyte differentiation independent of the activity of CEBPβ and CEBPδ^35^. In addition, GATA2/3 also inhibits PPARγ expression independent of the activity of CEBPβ and CEBPδ during adipogenesis^36^,^37^. In contrast, KLF5 activates PPARγ expression, induced early during adipocyte differentiation by CEBPβ and CEBPδ^38^. Here, we find that DPP8 and DPP9 are required for 3T3-L1 differentiation by targeting the expression of CEBPα and PPARγ. In our future directions, we plan to define whether the role of DPP8/9 on adipogenesis is dependent on CEBPβ and CEBPδ.

We noticed that a relatively high dose of P32/98 inhibitor (100 μM, 10 fold higher than IC50 value) is needed in the media to block differentiation in preadipocytes. Considering that DPP8 and DPP9 are cytosolic enzymes, the inhibitors need to pass the cell membrane to block the cytosolic activity of DPP8/9. We suggest it is, at least in part, the reason we need a high concentration of P32/98 to block the preadipocyte differentiation. High dose of DPP8/9 specific inhibitor 1G244 is used to block preadipocyte differentiation in our study as well. This is consistent with previous report, in which, 1G244 ameliorated DPP8/9 activity in HEK293 cells at high extracellular concentration of 8 μM (over 100 fold higher than IC50 value)^39^. In addition, non-inhibitory P34/98 is used as the control of P32/98. Moreover, we find that P34/98 had no effects on adipogenesis, which suggests minimal off-target effects.

A preclinical study^25^ has shown that the DPP8/9 inhibitor, allo-isoleucyl isoindoline derivative 4, resulted in severe toxicity, including death, alopecia and thrombocytopenia. In contrast to DPP4, which has an extracellular catalytic domain, DPP8 and DPP9 are cytosolic enzymes. A recent study found that allo-isoleucyl isoindoline derivative 4 is not able to penetrate the plasma membrane of mammalian cells, even at very high concentrations^39^. Therefore, the toxic effects observed with this inhibitor are not the result of DPP8/9 inhibition. Instead, it is most likely an “off-target” effect through an unknown mechanism outside the membrane. Moreover, a recent study found that high doses of vildagliptin, produced nearly complete inhibition of DPP8 and DPP9 enzyme activity *in vivo*, with no toxic side effects^40^. These findings indicate that the report of toxicity associated with certain DPP8/9 inhibitor compounds are not related to inhibition of DPP8 and DPP9. The DPP8/9 inhibitor 1G244 has an excellent membrane penetration capacity and rats treated with high dose of 1G244 do not exhibit pathological symptoms^39^. We observed that there was no difference dead cell counts with trypan blue staining compared to control, when 3T3-L1 cells were treated with 20μM 1G244.

Here we have found that DPP8 and DPP9 are required for preadipocyte differentiation. However, there is little information on the importance of DPP8 and DPP9 on *in vivo* adipogenesis. Since a knockout mouse model either for DPP8 or DPP9 is not available, the studies on role of DPPs *in vivo* mainly depend on the DPP inhibitors. There are five DPP4 inhibitors are currently in clinical use: saxagliptin^41^, sitagliptin^42^ and vildagliptin^43^, linagliptin^44^ and alogliptin^45^. From the literature, sitagliptin, saxagliptin, linagliptin and alogliptin have very less DPP8/9 inhibitory activity, whereas vildagliptin has quite potent activities against both DPP8 and DPP9^13^,^46^. However vildagliptin is the only DPP-4 inhibitor to show reduced fasting lipolysis that is associated with a reduction in fatter liver in patient^47^. Considering that DPP8 and DPP9 are cytosolic enzymes, the inhibitors need to pass the cell membrane to block the cytosolic activity of DPP8/9^39^. In our experiment, the concentration of inhibitors is needed at least 10 fold higher than IC50 value to block the adipognenesis in preadipocytes. So, the broad DPP inhibitors, like P32/98 and vildagliptin, are not perfect to study the role of DPP8/9 *in vivo*. Here we found that the DPP8/9 specific inhibitor 1G244 blocked the adipogenesis by inhibiting the expression of PPARγ during preadipocyte differentiation. TZDs (Rosiglitazone or troglitazone) were able to rescue the adipogenic defect in DPP8/9 inactivated preadipocytes. These data suggest that DPP8/9 inhibitor 1G244 has effects opposite to those of TZDs in preadipocytes. Considering the effects of TZDs on adipogenic and antidiabetic effects^8^, we hypothesize that the DPP8/9 specific inhibitor will have important role on adipogenesis and glucose homeostasis *in vivo*.

## Methods

### Materials

DPP8/9 inhibitor 1G244, DPP4 inhibitor MK-0431 and FAP inhibitor 3099 were a gift from Dr. Bachovchin (Tufts University, Boston, MA). P32/98 and P34/98 were received from Probiodrug^48^ (Halle, Germany). The PPARγ antibody was purchased from Cell signaling.

### Adipocyte differentiation

3T3-L1 cells were cultured to confluence in DMEM (Dulbecco’s modified Eagle’s medium) and supplemented with 10% calf serum. Two days after post-confluence (designated day 0), cells were induced to differentiate with a differentiation cocktail: DMEM supplemented with 10% fetal bovine serum (FBS), 1 μM dexamethasone (Sigma), 0.5 mM isobutylmethylxanthine (Sigma) and, 1 μg/ml insulin (Sigma) for 48 hours. The DPP inhibitors or PPARγ agonists were added to the differentiation cocktail as indicated. Then the media were replaced with DMEM supplemented with 10% FBS and 1μg/ml insulin for 48 hours. The cells were subsequently re-fed every 48 h with DMEM supplemented with 10% FBS. For 3T3-F442A differentiation: cells were induced to differentiate with 10 μg/ml insulin (Sigma) for 4 days. The DPP inhibitors were added together with insulin as indicated.

### Generation of stably transduced 3T3-L1 cells

One day prior to transfection, Phoenix Eco cells (NGVB, Indianapolis) were seeded at a density of 3 × 10^6^/10 cm dish. Retroviral vector was transfected into cells using TurboFectin 8.0 (Origene) according to the manufacturer’s instructions. Two days later, the medium containing viruses was filtered through 0.45 μm syringe filter and was used to infect 3T3-L1 cells. Two days after infection, cells were selected with 2.5 μg/ml puromycin or 6 ug/ml Blasticidine. For PPARγ2 overexpression experiment: pMSCVpuro- PPARγ2 was obtained from Addgene and control vector pMSCVpuro from Clontech. For DPP8 or DPP9 knockdown: DPP8 shRNA plasmid (Origene, TF504979) was puromycin resistance, with sequence 5′ TTCCTGAGTCTGGAGAACACTATGAACTG-3′ ; DPP9 shRNA plasmid (Origene, TG515456) was puromycin resistance, with sequence 5′- TGTCAAGCTGCGAGAAGGAACTGGTACAG-3′. For DPP8 and DPP9 double-knockdown: DPP9 shRNA sequence was constructed into blasticidine resistance vector pGFP-B-RS and co-transfected with puromycin resistance DPP9 shRNA plasmid.

### Reverse transcription and Real-time PCR

RNA was isolated using Roche RNA isolation kit and cDNA synthesized by iScriptcDNA synthesis kit (BioRad). Real-time PCR was performed using the CFX96 Touch™ real-time PCR detection system (BioRad) and the TaqMan PCR Reagent Kit with pre-designed primers and fluorescently labeled probes with the duplicates. The primers were from the Applied Biosystems: βactin, Mm00607939_s1; FABP4, Mm00445880_m1; Adiponectin, Mm00456425_m1; Leptin Mm00434759_m1; PPARγ2 Mm00440940_m1; CEBPα,Mm01265914_s1; CEBPβ, Mm00843434_s1; CEBPδ Mm00786711_s1; DPP4, Mm00494538_m1; DPP8, Mm00547049_m1; DPP9, Mm00841122_m1; FAP, Mm00484254_m1. The mRNA expression levels were calculated using the comparative CT method with b-actin as the endogenous reference gene, in accordance with the Applied Biosystems’ ABI PRISM, as described previously^49^.

### Oil red O staining

At the end of adipogenic differentiation, adipocytes were fixed in 4% paraformaldehyde (USB Corporation) for 8 min and were stained with Oil Red O for 30 min at room temperature. Subsequently, the cells were washed three times with PBS and imaged (Nikon, Melville, NY).

### Western Blot

For western blotting analysis, the cells were lysed in RIPA buffer (150 mmol/l NaCl, 1% Triton X-100, 0.5% sodium deoxycholate, 0.1% SDS, 50 mmol/l Tris-HCl, pH 7.4) containing phosphatase inhibitors and a protease inhibitor cocktail (Sigma). The lysate was then subjected to SDS-PAGE, transferred to PVDF membranes and incubated with the primary antibodies, followed by IRDye secondary antibody (Li-Cor). The bound antibody was visualized by Li-Cor Odyssey CLX system.

### DPP Activity

3T3-L1 cells were lysed in 0.1% Triton X-100. DPP activity was measured by luminescence, using DPPIV-Glo™ Protease Assay (Promega) according to the manufacturer’s instructions. The activity of individual DPPs was determined based on the differences between DPP activities with or without selective DPP inhibitors (10 μM).

### Statistical analysis

Every experiment has been repeated at least three times. Statistical analysis was performed using GraphPad software. Multiple comparisons were analyzed by One-way repeated measure ANOVA with post-hoc t-test using Dunnett’s method, and pair-wise comparison using Student’s t test. Results are presented as means ± SD, and a p value of <0.05 was considered statistically significant.

## Additional Information

**How to cite this article**: Han, R. *et al.* Inhibition of dipeptidyl peptidase 8/9 impairs preadipocyte differentiation. *Sci. Rep.* **5**, 12348; doi: 10.1038/srep12348 (2015).

## Supplementary Material

Supplementary Information
