# Supplementary material for: Inhibition of dipeptidyl peptidase 8/9 impairs preadipocyte differentiation
Source: Sci Rep. 2015 Aug 5;5:12348. doi: 10.1038/srep12348 (PMC4525143; doi:10.1038/srep12348)
Supplement: Supplementary Information [file srep12348-s1.pdf]

# Inhibition of dipeptidyl peptidase 8/9 impairs preadipocyte differentiation

Ruijun Han[1*], Xinying Wang[1*], William Bachovchin[2], Zofia Zukowska[1, 3] John W. Osborn[1]

1. Department of Integrative Biology and Physiology, University of Minnesota, Minneapolis, MN, USA

2. Sackler School of Biomedical Sciences, Tufts University School of Medicine, Boston, MA, USA.

3. Deceased in 2012

* These authors contributed equally to this work

Address correspondence to:

Ruijun Han, Ph.D.,
Email: rhan@umn.edu
Phone: 612-625-6255
Department of Integrative Biology and Physiology
University of Minnesota
3-137 CCRB
2231 6th St. SE
Minneapolis, MN 55455

**Supplementary figure 1** The DPP8/9 inhibitor blocks adipocyte differentiation in 3T3-f442A.

Oil Red O staining of control cells (DMI) and cells treated with 500µM DPP4 family inhibitor P32/98 (DMI+P32/98), 500µM inactive inhibitor P34/98 (DMI+P34/98), 20µM DPP4 inhibitor MK-0431(DMI+ MK-0431), 20µM DPP8/9 inhibitor 1G244 (DMI+1G244), 20µM FAP inhibitor 3099 (DMI+3099) at the day 8 of differentiation.

**Supplementary figure 2** DPP8 and DPP9 were the main forms of DPPs in 3T3-L1 cells. (A) DPP8, DPP9, DPP4 and FAP mRNAs were measured by real time PCR in 3T3-L1 cells at the differentiation day 2. (B) 3T3-L1 cells treated with DMI for 48 hours, the DPP activity was measured in cell lysate incubated with 10µM DPP4 inhibitor MK-0431(DMI+ MK-0431), 10µM DPP8/9 inhibitor 1G244 (DMI+1G244), 10µM FAP inhibitor 3099 (DMI+3099).

**Supplementary figure 3** DPP4 and FAP mRNA expression was decreased during 3T3-L1 differentiation. 3T3L1 cells were cultured in 10% FBS (NC) or treated with dexamethasone, isobutylmethylxanthine and insulin (DMI) from day 0 to day 2 during differentiation, mRNA levels of DPP4 and FAP were measured by real time PCR. β-actin expression was used as an internal control.

**Supplementary figure 4** (A) Knockdown of DPP8 and DPP9 mRNA by shRNA in 3T3-L1 cells. 3T3-L1 cells were infected with retrovirus containing shRNA plasmid (CTL shRNA), DPP8 shRNA plasmid (DPP8 shRNA), DPP9 shRNA plasmid (DPP9 shRNA) or both, then selected by puromycine or blasticidine. The expression of DPP8 and DPP9 was measured in these stable cells by real time PCR. (B) Knockdown of DPP8 by shRNA in 3T3-L1 detected by western blot.

**Supplementary figure 5** Inhibition of DPP8 and DPP9 prevents PPARγ1 induction during 3T3-L1 differentiation. PPARγ1 mRNA levels were measured after 48 hour treatment of dexamethasone, isobutylmethylxanthine and insulin (DMI) with or without the DPP4 inhibitor MK-0431(DMI+MK-0431), the DPP8/9 inhibitor 1G244 (DMI+1G244), the FAP inhibitor 3099 (DMI+3099).

**Supplementary figure 6** Full length blots of Figure 5A

**Supplementary figure 7** Full length blots of Figure 4B

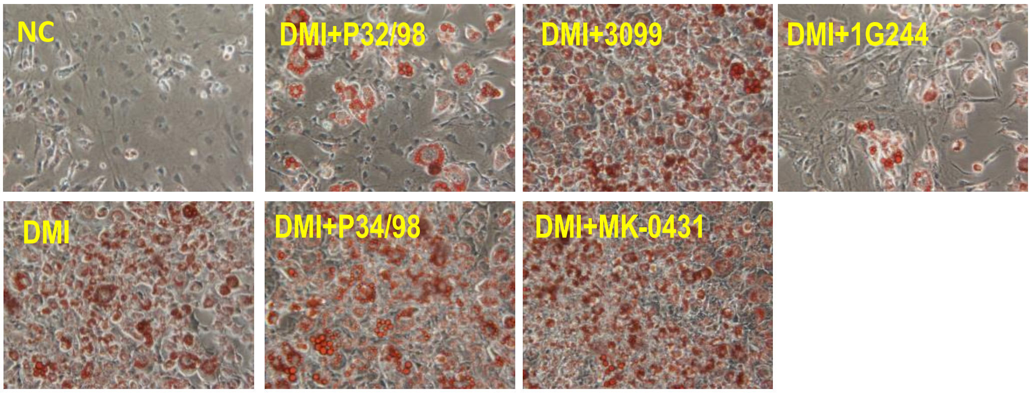


**Supplementary figure 1**

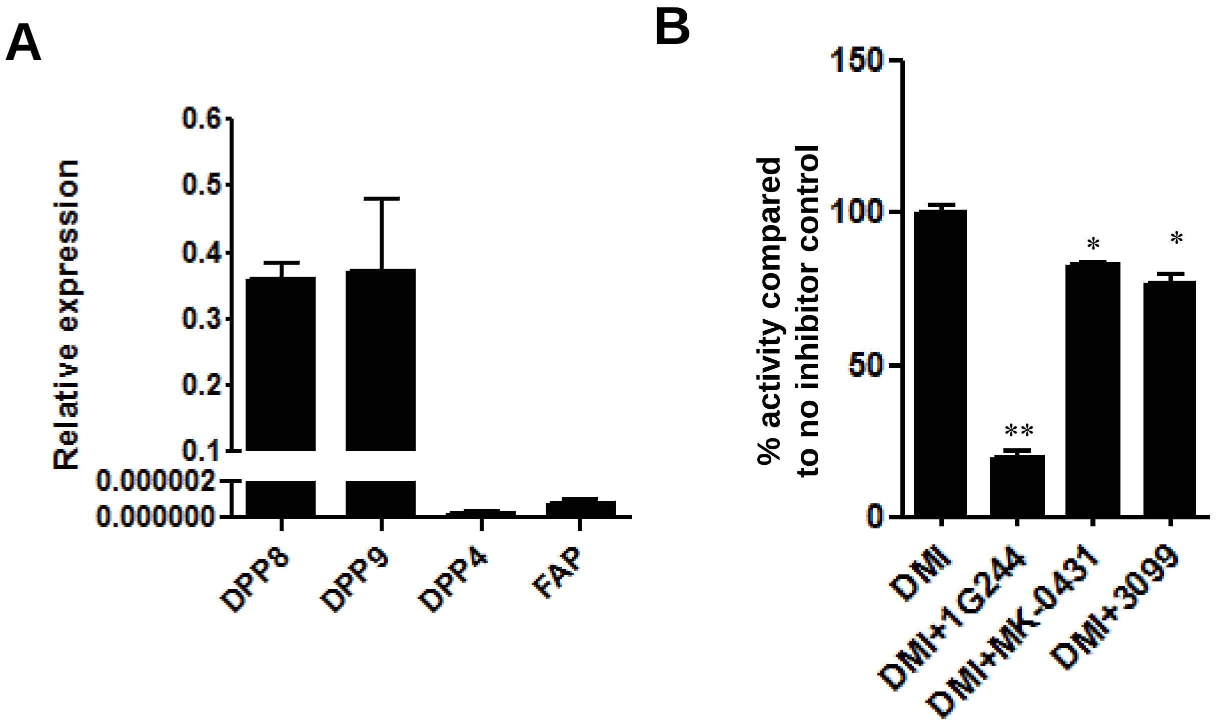


**Supplementary figure 2**

## DPP 4

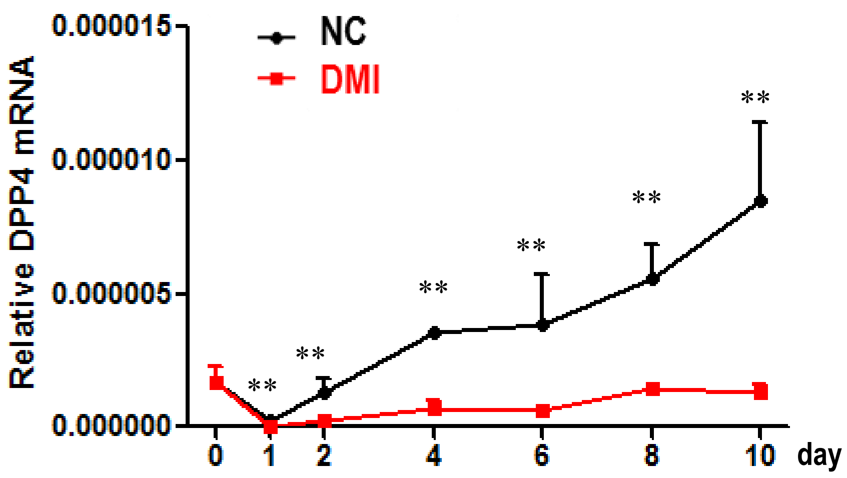


## FAP

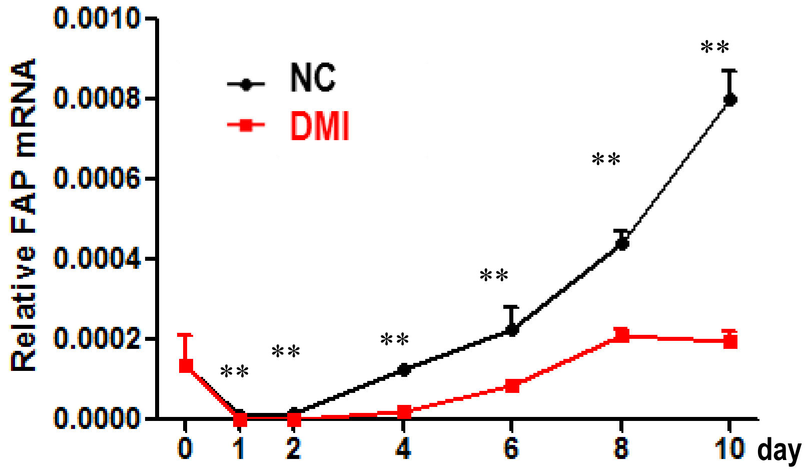


**Supplementary figure 3**

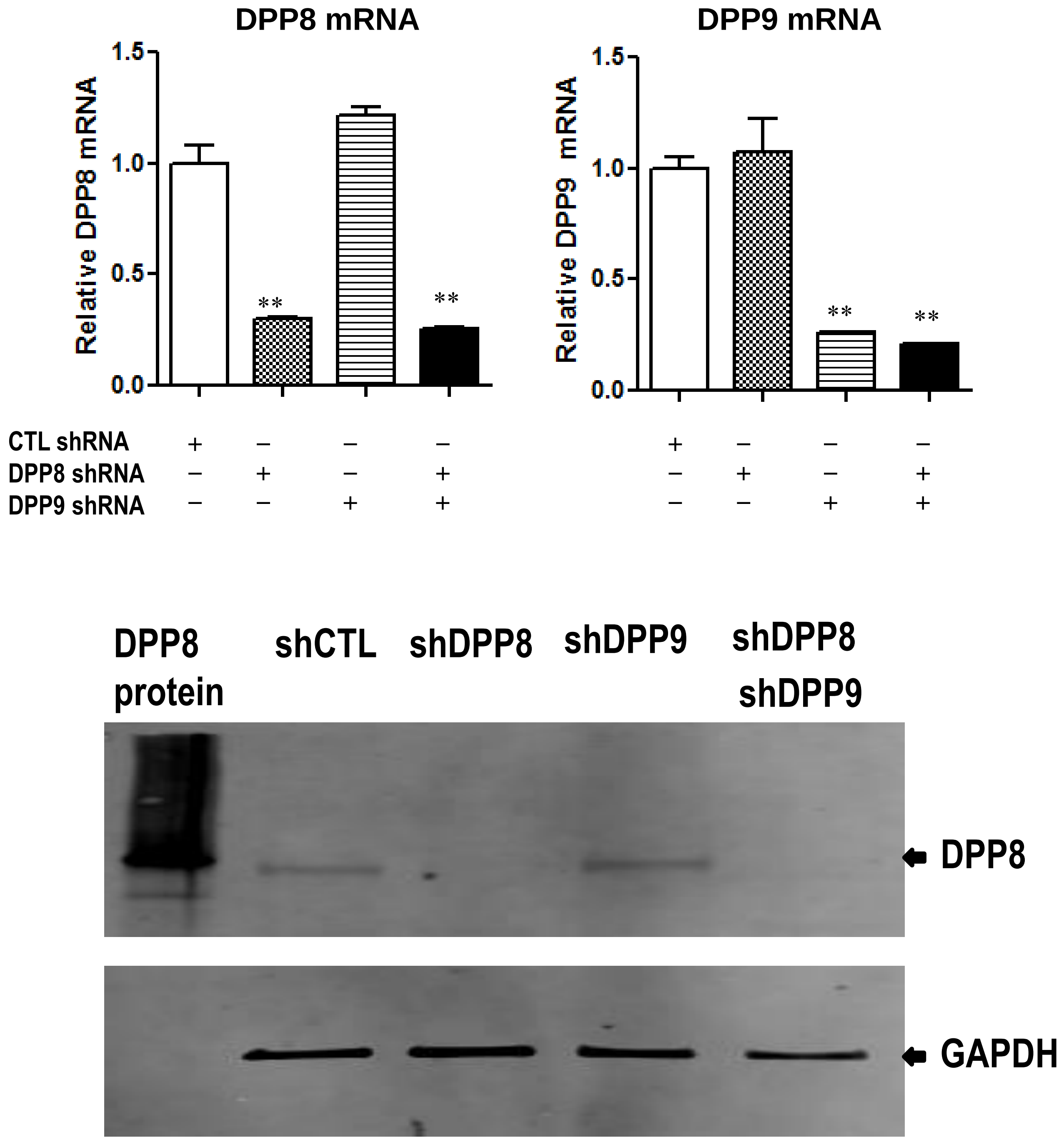


**Supplementary figure 4**

**PPARγ1**

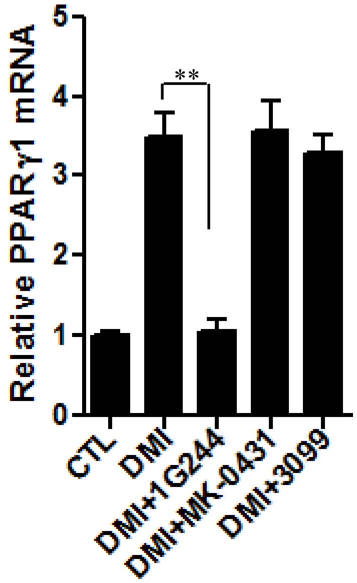


**Supplementary figure 5**

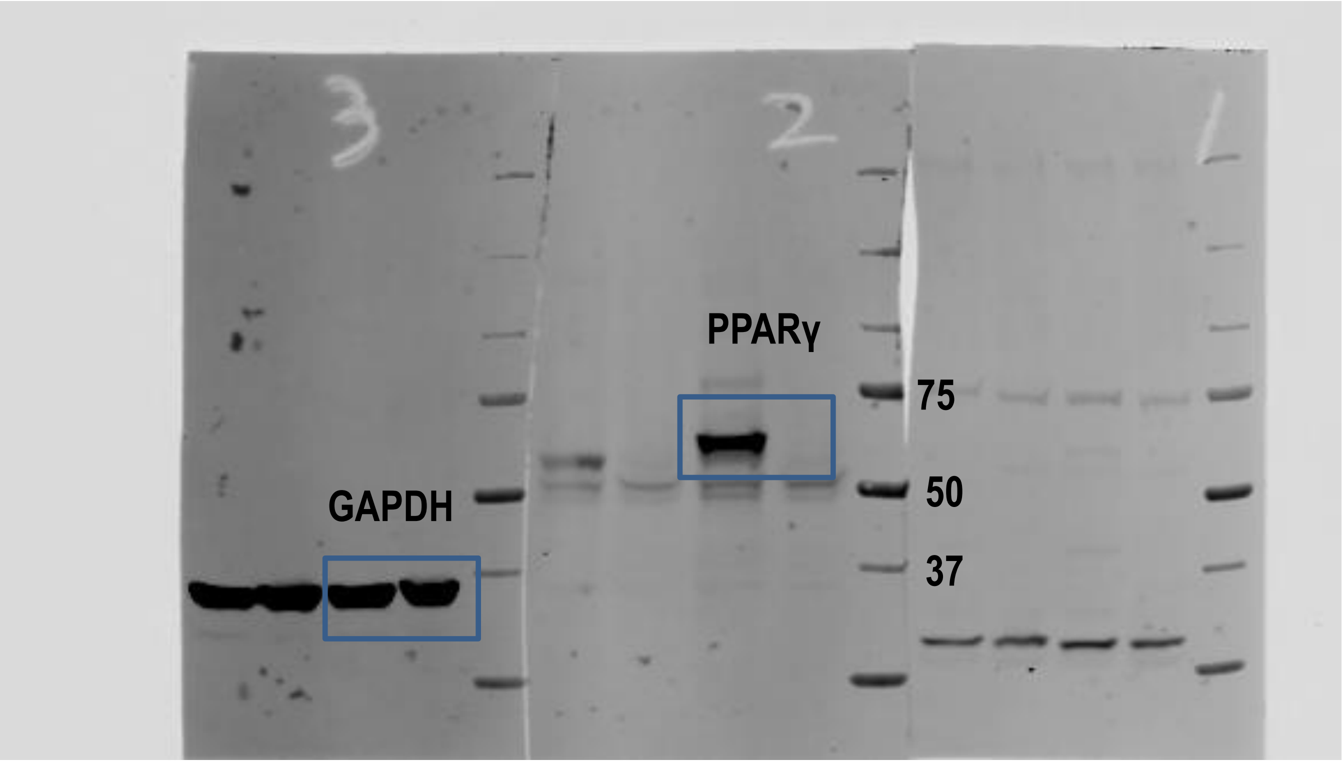


**Supplementary figure 6**

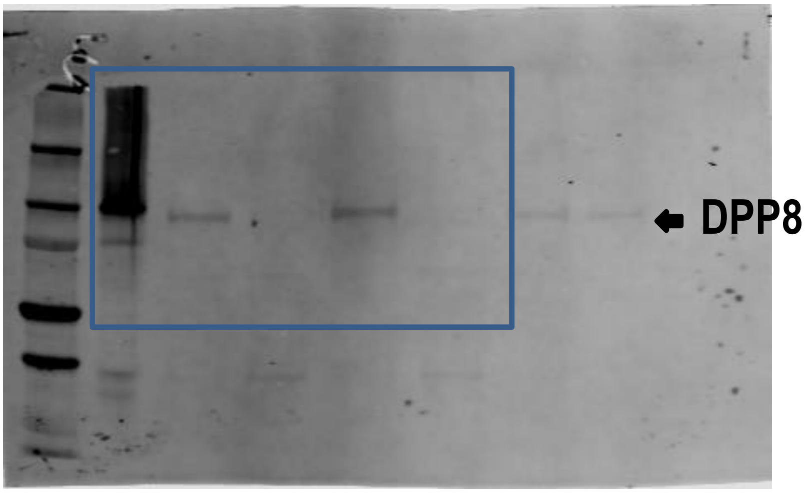


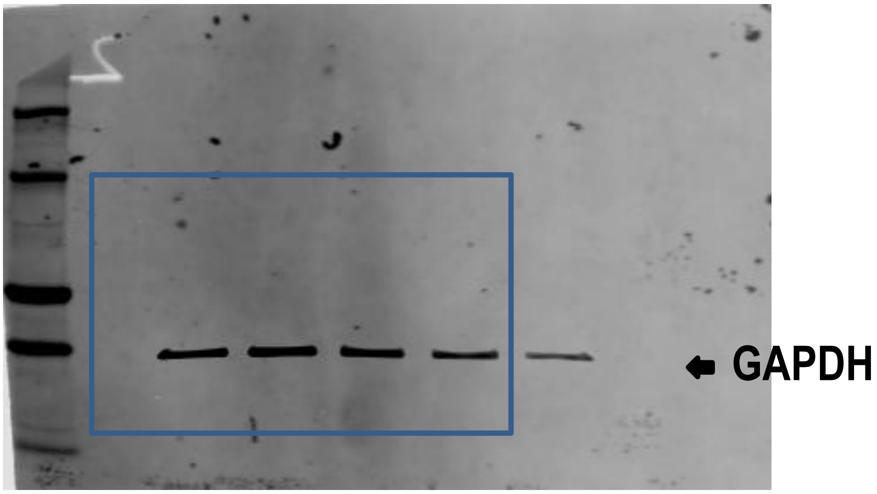


**Supplementary figure 7**
